# Patient safety subcultures among registered nurses and nurse assistants in Swedish hospital care: a qualitative study

**DOI:** 10.1186/s12912-014-0039-5

**Published:** 2014-11-26

**Authors:** Marita Danielsson, Per Nilsen, Annica Öhrn, Hans Rutberg, Jenni Fock, Siw Carlfjord

**Affiliations:** Department of Medical and Health Sciences, Division of Health Care Analysis, Linköping University, Linköping, SE- 581 83 Sweden; Department of Health and Development, County Council of Östergötland, Linköping, SE- 581 91 Sweden; Department of Medical and Health Sciences, Division of Community Medicine, Linköping University, Linköping, SE- 581 83 Sweden

**Keywords:** Nurses, Patient safety, Safety culture, Qualitative research

## Abstract

**Background:**

Patient safety culture emerges from the shared assumptions, values and norms of members of a health care organization, unit, team or other group with regard to practices that directly or indirectly influence patient safety. It has been argued that organizational culture is an amalgamation of many cultures, and that subcultures should be studied to develop a deeper understanding of an organization’s culture. The aim of this study was to explore subcultures among registered nurses and nurse assistants in Sweden in terms of their assumptions, values and norms with regard to practices associated with patient safety.

**Methods:**

The study employed an exploratory design using a qualitative method, and was conducted at two hospitals in southeast Sweden. Seven focus group interviews and two individual interviews were conducted with registered nurses and seven focus group interviews and one individual interview were conducted with nurse assistants. Manifest content analysis was used for the analysis.

**Results:**

Seven patient safety culture domains (i.e. categories of assumptions, values and norms) that included practices associated with patient safety were found: responsibility, competence, cooperation, communication, work environment, management and routines. The domains corresponded with three system levels: individual, interpersonal and organizational levels. The seven domains consisted of 16 subcategories that expressed different aspects of the registered nurses and assistants nurses’ patient safety culture. Half of these subcategories were shared.

**Conclusions:**

Registered nurses and nurse assistants in Sweden differ considerably with regard to patient safety subcultures. The results imply that, in order to improve patient safety culture, efforts must be tailored to both registered nurses’ and nurse assistants’ patient safety-related assumptions, values and norms. Such efforts must also take into account different system levels. The results of the present study could be useful to facilitate discussions about patient safety within and between different professional groups.

## Background

Patient safety has been widely reported and discussed since the report *To Err Is Human* [1]. The report put the issue high on the agenda for managers, practitioners and policy makers in health care as well as for the general public. In the decade after the report, concerted efforts to improve safety in health care have been launched in many countries and patient safety has emerged as a vital research field. Sources of safety and risks can be found at all levels of the system, the individual, interpersonal and organizational levels, and the importance of a systems approach to understand interacting influences on patient safety has been emphasized [2–4].

Patient safety culture has become an important focus of patient safety research and there is emerging evidence to support the potential effectiveness of interventions aimed at improving safety culture. The best evidence to date includes strategies comprising multiple components that incorporate team training and mechanisms to support team communication and include management engagement in front-line safety work [5]. There is a lack of conceptual consensus concerning the definition of patient safety culture. The concept is frequently confused with patient safety climate; the two concepts overlap and the terms are often used interchangeably. A general agreement, however, is that patient safety culture emerges from the shared assumptions (unspoken beliefs and expectations), values (important and lasting ideals and beliefs) and norms (beliefs about how members of a group should behave in a given context) among members of an organization, unit or team with regard to practices that directly or indirectly influence patient safety. This sometimes is expressed as “the way things are done around here” [6,7].

Patient safety culture is a subset of organizational culture, and includes those parts of the organizational culture that influence patient safety [8]. Researchers have increasingly argued that an organization’s culture is an amalgamation of many cultures, and that subcultures should be studied to develop a deeper understanding of an organization’s culture [9]. Studies suggest that patient safety cultures can differ between departments, specialties and professional groups [10–13].

The existence of professional subcultures in health care has been demonstrated in studies of health care organizations [14–16] and implementation of reforms and methods [17,18]. Subcultures in health care are potentially important from a patient safety point of view because patient safety might be at risk if subcultures are not aligned with the organization-wide safety goals or if they hinder effective teamwork. However, few studies have examined professional patient safety subcultures, and researchers have called for more qualitative research to obtain improved understanding of patient safety culture and subcultures [19,20].

Nurses constitute the largest personnel category in health care in Sweden [21]. The importance of nurse staffing and education in achieving safe patient care has been emphasised in several studies, most recently by Aiken et al. [22] and Ball et al. [23]. Modern care is increasingly characterized by multi-professional teamwork and the team closest to the patient usually consists of registered nurses and nurse assistants (non-registered nursing staff). Nurse assistants typically work under registered nurses’ supervision and play a key role in keeping the nurses up to date on vital information about the patients’ conditions. Registered nurses and nurse assistants in Sweden differ with regard to their level of education and work duties. Considering the critical role of these groups, the aim of this study was to explore subcultures among registered nurses and nurse assistants in Sweden in terms of their assumptions, values and norms with regard to practices associated with patient safety.

## Methods

The study employed an exploratory design and used a qualitative method with focus group interviews and individual interviews to gather data. The study population consisted of registered nurses and nurse assistants from medical and surgical wards at one university hospital (600 beds, about 5500 employees) and one county hospital (310 beds, about 2200 employees) in southeast Sweden. The number of employed registered nurses at the wards varied from 14 to 70 and nurse assistants from 13 to 50. Focus group interviews are well suited to explore group practices, interactions and norms, suggesting that the method is suitable to assemble data concerning patient safety culture [24]. To obtain a satisfactory amount of information and allow for exploration of differences, four focus group interviews with registered nurses and four with nurse assistants at each hospital were planned, as suggested by Kreuger and Casey [24].

### Data collection

To recruit informants for the study, we sent information to the manager of each ward with a request to invite four to six registered nurses and four to six nurse assistants for an interview on patient safety. To be included, informants should have been employed permanently for at least six months and work 20 hours per week or more. The individuals who volunteered to participate were then individually informed by e-mail about the aim and contents of the study. Showing up at the interview session was interpreted as informed consent. A description of the informants’ characteristics can be found in Table 1.

**Table 1:** **Characteristics of the informants**

**Characteristics**	**Registered nurses (** ***N*** **= 28)**	**Nurse assistants (** ***N*** **= 24)**
Sex, *n* (%):		
Male	2 (7)	1 (4)
Female	26 (93)	23 (96)
Age range (years)	24–57	22–62
Average age (years)	36.6	44.3
Years of practice, *n* (%)		
0,5–3 years	14 (50)	6 (25)
4–10 years	8 (29)	5 (21)
11–15 years	4 (14)	3 (13)
16–20 years	0	0
21 years or more	2 (7)	10 (42)

A semi-structured interview guide was prepared by the research team. The guide consisted of themes concerning assumptions, values and norms related to patient safety, drawing on inspiration from questions posed in Walk Rounds, as described by Frankel [25]. After a pilot focus group interview (not included in the analysis), minor changes were made to the interview guide and an introductory question was added [24]. After the introductory question, “what is patient safety and what does it mean to you?” the interview focused on (a) perceptions of responsibility, (b) situations where mistakes are made, and (c) concerns or worries about patient care.

The first author (MD) served as moderator and the interviews were observed by an assistant taking notes. MD has experience in conducting focus group interviews and works part-time as a national coordinator of patient safety culture issues for Swedish Association of Local Authorities and Regions. After each interview the moderator and the assistant had a brief talk about their impressions, in line with recommendations by Krueger [24].

All interviews were conducted between April and August 2012. Table 2 provides an overview of the interviews. Individual interviews were conducted only when the work situation did not permit more than one person to leave the ward. All interviews took place in a room near the ward where the study informants worked and lasted between 20 and 45 minutes.

**Table 2:** **Overview of the interview informants**

**Registered nurses**					**Nurse assistants**				
**Interview number**	**Hospital** ^**1**^	**Ward** ^**2**^	**Interviewtype** ^**3**^	**Number of informants**	**Interview number**	**Hospital** ^**1**^	**Ward** ^**2**^	**Interview type** ^**3**^	**Number of informants**
1	U	M	F	4	10	U	M	F	3
2	C	M	F	4	11	C	M	F	3
3	U	S	F	3	12	U	S	F	3
4	C	S	F	2	13	C	S	F	4
5	U	M	F	4	14	U	M	F	4
6	C	M	F	3	15	C	M	F	2
7	U	S	I	1	16	U	S	I	1
8	U	S	I	1	17	C	S	F	4
9	C	S	F	6					

^1^U = University hospital, C = County hospital.

^2^M = Medical ward S = Surgical ward.

^3^F = Focus group interview, I = Individual interview.

### Data analysis

The interviews were recorded and then transcribed verbatim. The data were inductively analysed using manifest content analysis, which is a structured technique for coding and categorizing empirical data in an explorative and descriptive way [26,27]. The analysis was conducted in several steps with the aim of identifying assumptions, values and norms with regard to practices associated with patient safety. The first author (MD) listened to the recordings to ensure that the transcripts were accurate. Then three of the other authors (SC, JF, PN) read the transcripts to obtain an understanding of the content. Meaning units were identified, first individually by the four authors (MD, SC, JF, PN), then several sessions were held to compare and discuss the meaning units. The meaning units were condensed and labelled with codes. The condensed meaning units were combined into subcategories based on similarity of content. Discussions among the four authors continued until consensus was reached [28] to prevent researcher bias and strengthen the internal validity*.* Categories were formed on the basis of similarity of the content of the subcategories. Quotations were identified to report the findings and illustrate the content.

### Ethics

Ethical approval was received for the study by the regional Ethical Review Board at Linköping University, Sweden (Dnr 2012/23-31). Participation was voluntary and the informants could interrupt participation at any time. Confidentiality regarding the collected data was assured.

## Results

**Figure 1:**
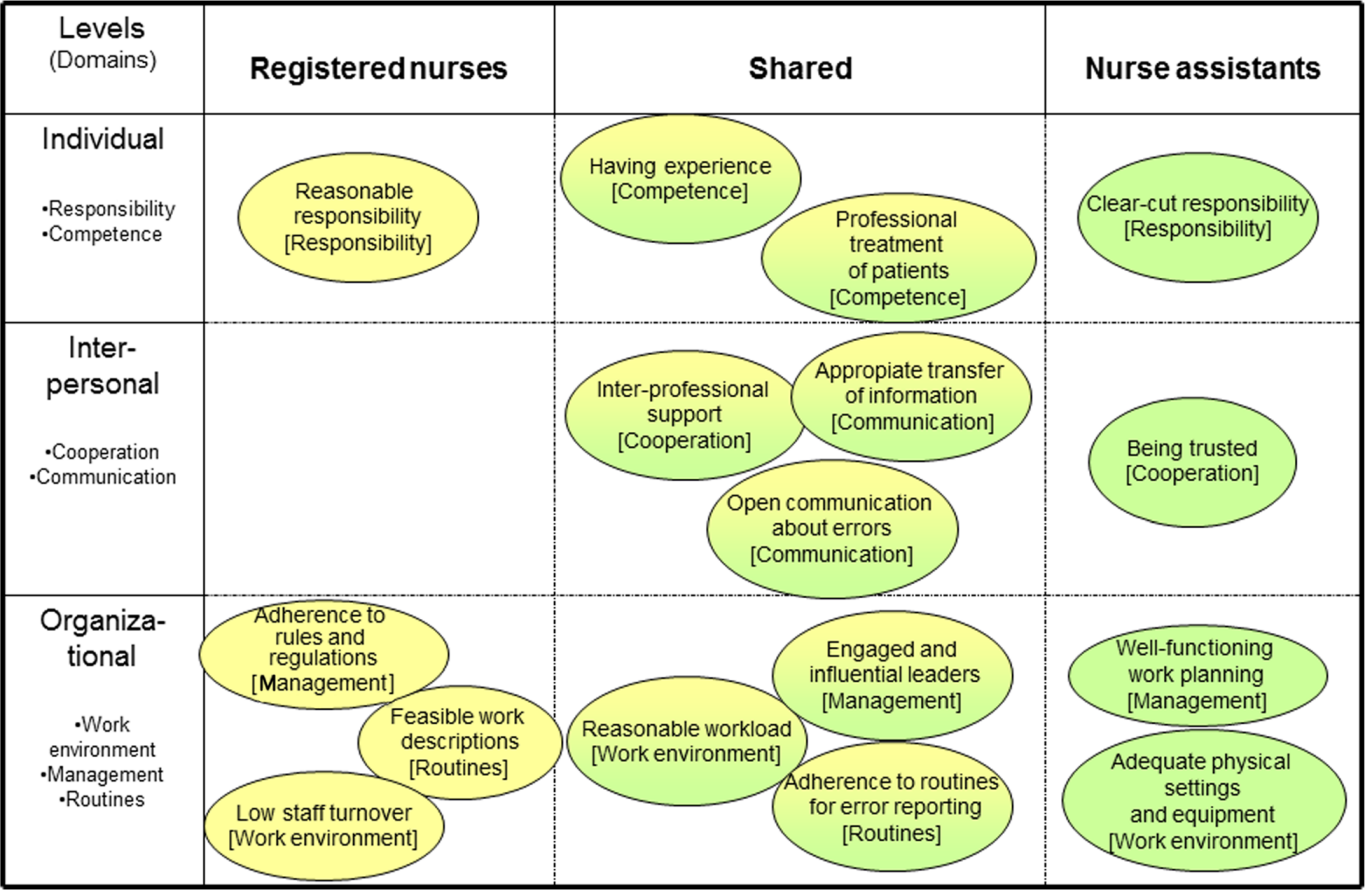
**Subcategories pertaining to registered nurse and nurse assistants, sorted according to domains and system levels.**

Analysis of the data yielded seven categories, hereafter referred to as domains, comprising 16 subcategories expressing assumptions, values and norms with regard to practices associated with patient safety. The seven domains corresponded with three overarching levels: individual, interpersonal and organizational (Figure 1). Some of the subcategories were relevant only for registered nurses, others only for nurse assistants, and some for both groups. Subcategories were labelled to indicate “desirable” assumptions, values and norms related to patient safety even though the informants sometimes expressed the “opposite”, e.g. perceptions of not being trusted or not having reasonable responsibility (Figure 1).

In the following presentation of the findings, […] indicates that words were omitted from the quotation, author comments to clarify quotations appear in brackets [ ], and hesitation is indicated by ….. The numbers identify which interview the quote was excerpted from, as displayed in Table 2.

### Individual level

#### Responsibility

##### Reasonable responsibility

Registered nurses expressed that having a reasonable level of responsibility was important for patient safety. They felt that their responsibilities could be too extensive and could encompass some responsibilities legitimately belonging to physicians. Although some tasks could be delegated to nurse assistants, the registered nurses assumed responsibility for such tasks anyway.

*Physicians ask us, ‘Is this correct, what do you think about it?’ It’s good that they do [ask us], but I don’t want to take responsibility for the dose that the physician should prescribe. It’s not my area [of competence] even though I’ve seen many prescriptions.* (RN 1)

##### Clear-cut responsibility

The nurse assistants perceived that many of their responsibilities were unspecified. This caused frustration and might affect patient safety culture negatively. To some extent, they felt they did not have specific tasks.

*Of course we have a responsibility towards the patient. But I cannot think of anything really specific [responsibility] for us.* (NA 17)

#### Competence

##### Having experience

Both registered nurses and nurse assistants stated that having experience was important for patient safety. Experienced colleagues conveyed a sense of security and could be asked for advice in difficult situations. They believed that certain capabilities and skills could not be learnt without having worked for an extended period of time.

*You get better and better at seeing changes [concerning the patient’s condition]. Early in your career you don’t notice these small signs because there are so many other things you must learn. Colleagues with experience can teach you what to look for.* (NA 11)

Working with less experienced colleagues sometimes felt unsafe. For example, a registered nurse mentioned that inexperienced registered nurses do not respond to inaccurate prescriptions.

*It’s dangerous when there are many new [registered] nurses. They do not react to [erroneous] unreasonable prescriptions.* (RN 1)

##### Professional treatment of patients

Registered nurses and nurse assistants both highlighted the importance of professional treatment of patients for patient safety. Treating patients professionally engenders a sense of mutual trust, which was perceived conducive to patient safety. The ideal was to always remain calm with the patients even in stressful situations.

*You can leave [the ward] to find out about something and then return, but it’s important not to show hesitation in front of the patient. You should give a secure impression.* (RN 8)

They also expressed that the focus should always be on the interaction with the patient.

*As a patient, you have to feel secure, that you are being treated by professionals who know their skills and that you are in safe hands. The patients are calmer if they feel they can depend on us.* (NA 13)

### Interpersonal level

#### Cooperation

##### Interprofessional support

The registered nurses and nurse assistants described the importance of support from other professions for patient safety.

*The physicians have an incredible [amount of] trust in us.* (RN 9)

The registered nurses described having a great deal of support from the physicians, but they felt it did not always exist to the extent that they wanted.

*There are physicians in the clinic from whom I would never accept a verbal prescription, never […] because it [the prescriptions] can change many times before it gets into print.* (RN 2)

Interprofessional support between physicians and registered nurses contributed to creating an open climate where the nurses felt they could ask physicians without disturbing them. However, the nurse assistants did not always feel they were supported by the registered nurses. They expressed frustration about being unable to care for patients because they felt obligated to attend to other matters, which affected the climate of cooperation.

*You have to do everything else too [except caring for the patients]. The cleaning, kitchen […] it’s rarely a registered nurse doing these things.* (NA 16)

##### Being trusted

The nurse assistants described feelings of not being trusted, which they believed could have a negative impact on patient safety.

*Sometimes you are invisible. It’s a common feeling.* (NA 12)

*If you say that you have seen something, no one listens. But when the registered nurse says the same thing, then it will be heard. And this annoys me very much.* (NA 12)

#### Communication

##### Open communication about errors

Both registered nurses and nurse assistants stressed the importance for patient safety of talking openly about errors despite the difficulties involved in order to learn from mistakes.

*Some may want to keep it to themselves, but I think its good talking about it [an error]. You might get some support, ‘This has happened to me too’. Otherwise you feel like a failure.* (RN 3)

*You don’t want to feel uncomfortable in front of your colleagues. … It is not always acceptable to say something … you get criticized.* (NA 14)

*A situation that I and a registered nurse were involved in …we have talked about it several times. We have learned some; what has happened has happened, but we learn from the experience.* (NA 16)

##### Appropriate transfer of information

Both groups expressed a belief that transfer of verbal and written information is important to patient safety. They viewed communication as a risk area because information was not always transferred correctly. The registered nurses particularly emphasized the importance of appropriate information transfer among registered nurses and between registered nurses and physicians.

*Information transfer is difficult. You see [problems with] it every day - complex patients arrive and you get no information.* (RN 5)

*Communication is also important between professions; for example, physicians should explain the plan. If we know the plan, we don’t need to spend so much time calling the physician.* (RN 4)

The nurse assistants described inappropriate transfer of information.

*We’ve talked about documenting more. We do not really write much in the medical record… It’s usually us who give [the patient] a catheter but in the medical record it says that the registered nurse did it.* (NA 14)

### Organizational level

#### Work environment

##### Reasonable workload

Members from both groups stated that workload was associated with patient safety. They experienced shortage of time for patients, stress and tiredness. They perceived the worst situations occurred at night and at the weekend*.* Some of the registered nurses complained that they felt obliged to do many things beyond taking care of patients.

*Patient safety is when you cancel your own lunch … Though I do not know if it will be safe for the patient when this happens […] that’s a hazard in itself. Stuff like that makes you finally so tired that you might make a mistake you never thought you could do.* (RN 9)

*Lack of time, you lose control … and then finally you can’t stand it, you can’t handle it anymore.* (NA 15)

##### Low staff turnover

Registered nurses discussed staff turnover as an important factor that could affect patient safety. They talked about the risks of having a high staff turnover, which they felt could have a detrimental effect on the competence and the relationships of the staff.

*Recently many registered nurses have left… because they have felt that there is too much pressure. We have a lot of responsibility and sometimes you can’t cope.* (RN 5)

##### Adequate physical settings and equipment

Nurse assistants perceived that work conditions in terms of having functional physical settings and proximity to equipment were important for patient safety.

*[It’s important] to have premises adapted for the activities …. Our kitchen is located far away. It takes time for us to get there, which means we have less time for the patients. It feels as if we are leaving the ward when we go to get a yogurt [for the patient].* (NA 10)

#### Management

##### Engaged and influential leaders

Both groups discussed the importance of having engaged leaders to achieve patient safety. The registered nurses expressed an ideal of having leaders who assume responsibility for staffing and provide support. The nurse assistants talked about the importance of leaders who were supportive, fair and encouraging.

*If you say ‘It is too stressful on the ward’, they [the management] answer, It’s your responsibility to get it done. That’s the way it is, make it as good as possible.* (RN 9)

*I think we have very good support and encouragement from our manager, it feels like she wants to help us but she can’t do much about it [the staffing situation].* (RN 1)

##### Adherence to rules and regulations

The registered nurses perceived that management adherence to rules and regulations concerning the staff situation were important for patient safety. Short-term problems lead to changes in the local staffing rules. For example, a rule stating that a nurse with less than 2 years of experience is not allowed to work nights is not adhered to by the management when there are staff shortages, which could jeopardize patient safety.

*Earlier we had a rule that you should not work nights in the first two years because it required too much and we work very much alone during nights....* (RN 5)

*But you do not really have the option to say ‘no’ when you’ve worked a year … There is always a shortage of those who have the emergency competencies.* (RN 1)

##### Well-functioning work planning

The nurse assistants expressed the importance of having functioning work planning from the management for patient safety.

*We have a staffing unit, but the problem is that they open 8 am and we start our work 6:45 am […] Therefore it’s not possible to get additional staff during a busy morning; that’s a patient safety risk.* (NA 14)

#### Routines

##### Adherence to routines for error reporting

Both groups said that the norm was to report when errors occurred, something they believed could affect patient safety. However, they pointed out that reporting was inconsistent; errors in some areas were reported more frequently.

*It’s obvious that you have to write an error report [when something has happened] and perhaps consider what could have been done better, so you are more prepared the next time it happens.* (NA 11)

##### Feasible work descriptions

The registered nurses believed that having written work descriptions is important for patient safety, but having too many written instructions could make it difficult to keep up, leading to problems with adherence.

*We hardly have any routines, for how we should work for the best results. Nothing is written down, so we work like we have always have done.* (RN 5)

*The problem is that we have a lot of routines here […] But may be 80% of our error reporting concerns deviations from routines.* (RN 7)

## Discussion

This study sought to explore patient safety subcultures among registered nurses and nurse assistants in Sweden. We identified considerable differences in the patient safety culture of the two groups. Of the 16 subcategories, only half were shared by the two groups. These findings are consistent with research that suggests that health care organizations comprise disparate professional subcultures that are only partially overlapping [29].

We identified seven patient safety culture domains concerning practices associated with patient safety corresponding with three systems levels: individual, interpersonal and organizational. Although culture is a social phenomenon, the most basic unit is the individual because the individual either replicates and reinforces or alters and modifies the assumptions, values and norms that form the culture. Our findings underscore the relevance of applying a systems perspective to patient safety culture [2–4].

The two domains at the individual level identified in our study, responsibility and competence, are not clearly addressed in commonly used instruments for measuring patient safety culture. The other five domains (cooperation, communication, work environment, management and routines) are represented in the three most recommended instruments for measuring patient safety culture [30–33]. Norms and values concerning responsibility and competence among registered nurses and nurse assistants may affect the safety culture, and could possibly contribute to a more complete picture if included.

Responsibility emerged in our study as an important aspect of the patient safety culture, but was viewed differently by the two groups. The registered nurses called for a reasonable level of individual responsibility, expressing that they had difficulties setting the limits for their responsibilities. Studies have shown that registered nurses struggle with responsibility and feelings of being burdened [34,35]. In contrast, the nurse assistants viewed responsibility in terms of being vague and, in some cases, they expressed that they were not given sufficient responsibility for important tasks. Issues regarding responsibility might influence the patient safety culture.

In our study, competence was described in terms of having experience and providing professional patient treatment. According to the informants, competence develops over time and a low staff turnover was described as conducive to developing competence. Research indicates that low staff turnover is indeed important for patient safety [22,36,37]. According to our findings the close relationship between low staff turnover and patient safety could be explained by team competence building over time, also including the development of a patient safety culture.

Regarding the interpersonal level, communication was perceived as important by the informants from both groups. Communication problems are a well-known risk area in health care [38,39]. Previous research in Sweden has shown that nurse assistants communicate predominantly with other nurse assistants, whereas registered nurses communicate more broadly, with different professional groups [40]. Other research also suggests that professional boundaries can inhibit communication between different professional groups in health care [41]. In our study, both registered nurses and nurse assistants believed that the communication related to information about patients and communication about failure could be developed in order to improve patient safety.

Another finding at the interpersonal level concerned cooperation. The nurse assistants sometimes felt that they were not being fully trusted by the other professions. Considering that nurse assistants constitute the second largest professional group in Swedish health care, representing 27 per cent of the workforce [21], it seems to be important to address the issue. Trust has been shown to be crucial for team performance [42]. Not being trusted might undermine the individuals’ attitudes regarding patient safety.

Three of the domains concerned the organizational level: routines, management and work environment. The informants expressed the value of well-functioning routines, managers who are adherent to rules and appropriate work environments in terms of sufficient staffing. Adherence to routines for error reporting seems to be well established among registered nurses and nurse assistants [43], which indicate that organizational learning has occurred. Regarding management, leadership support has been found to be one of the most important factors for a positive safety culture [19]. When managers deviate from rules, as was mentioned by our informants, it is interpreted negatively, and might undermine the safety culture among staff. Both registered nurses and nurse assistants perceive that a heavy workload influences patient safety culture negatively, also affecting patient safety. This is supported by findings published by Aiken et al. [22].

Our findings suggest that the assumption that health care organizations are characterized by a single dominant patient safety culture represents a simplification of this complex concept. The study points to the need for more research into the assumptions, values and norms with regard to patient safety practices among different professional groups in health care. Interventions to improve patient safety have to be tailored and directed at all levels according to the prevailing safety subcultures of specific professional groups. The possibility to discuss patient safety issues across professional boarders, and with managers fully engaged in the issue, might result in increased convergence of professional groups’ assumptions, values and norms concerning practices that influence patient safety, thus potentially reducing differences between subcultures. It is important to further explore potential and actual consequences of differences between professional subcultures in health care.

### Methodological considerations

This study has some limitations that should be considered when interpreting the findings. The voluntary nature of participation in the study means that the study sample may differ from the broader population of registered nurses and nurse assistants in their assumptions, values and norms related to patient safety practices. All qualitative research is limited regarding its relevance and generalizability to other settings and populations. The patient safety culture domains and subcategories in this study pertaining to the two professional groups should not be interpreted as an exhaustive list of all possible assumptions, values and norms. Other studies may yield different factors or give different priorities to various factors.

One limitation is that two of the focus groups consisted of only two informants. When four or more informants accepted participation in a focus group, we went ahead with the interview even if only two people showed up. All interviews were included in the analysis.

To strengthen the content validity several discussions were held between four of the authors during the analysis process. Different professions and experiences among the authors were perceived as valuable in the analysis process.

## Conclusions

Registered nurses and nurse assistants in Sweden differ considerably with regard to patient safety subcultures, i.e. their assumptions, values and norms with regard to practices that influence patient safety. Well-functioning routines, managers who adhere to rules and appropriate work environments were valued organizational-level factors. Communication about patients and errors was considered important at the interpersonal level. Adequate responsibility and competence were highlighted at the individual level.

The results imply that, in order to improve patient safety culture, efforts must be tailored to both registered nurses’ and nurse assistants’ patient safety-related assumptions, values and norms. Such efforts must also take into account different system levels. The results of the present study could be useful to facilitate discussions about patient safety within and between different professional groups.
